# The Restoration of Vitamin D Levels Slows the Progression of Renal Ischemic Injury in Rats Previously Deficient in Vitamin D

**DOI:** 10.3389/fmed.2021.625647

**Published:** 2021-04-01

**Authors:** Michele Santiago dos Santos, Daniele Canale, Desiree Rita Denelle Bernardo, Maria Heloisa Massola Shimizu, Antonio Carlos Seguro, Rildo Aparecido Volpini, Ana Carolina de Bragança

**Affiliations:** ^1^Laboratorio de Investigacao Medica 12 (LIM12), Faculdade de Medicina, Universidade de São Paulo, São Paulo, Brazil; ^2^Laboratorio de Investigacao Medica 12 (LIM12), Hospital das Clinicas HCFMUSP, Faculdade de Medicina, Universidade de São Paulo, São Paulo, Brazil

**Keywords:** kidney disease progression, ischemia/reperfusion injury, vitamin D deficiency, vitamin D replacement, inflammation, fibrosis, experimental model

## Abstract

Chronic kidney disease (CKD) remains a global public health problem. The initial damage after ischemia/reperfusion (I/R) injury plays an important role in the pathogenesis of acute kidney injury (AKI) and predisposition to CKD. Several studies have been showing that *nontraditional* risk factors such as AKI and hypovitaminosis D could also be involved in CKD progression. Vitamin D deficiency (VDD) is associated with hemodynamic changes, activation of inflammatory pathways and renal disease progression (RDP) following I/R-AKI. Strategies for prevention and/or slowing RDP have been determined and the sufficiency of vitamin D has been emerging as a renoprotective factor in many diseases. Therefore, we investigated the effect of the restoration of vitamin D levels in the progression of I/R injury (IRI) in rats previously deficient in vitamin D. On day 30, male Wistar rats were submitted to bilateral 45 min IRI and divided into three groups: IRI, standard diet for 120 days; VDD+IRI, vitamin D-free diet for 120 days; and VDD+IRI+R, vitamin D-free diet in the first 30 days and just after I/R, we reintroduced the standard diet in the last 90 days. After the 120-day protocol, VDD+IRI+R rats presented an improvement in the renal function and renal protein handling followed by a smaller fractional interstitial area. Furthermore, those animals exhibited a reestablishment regarding the hemodynamic parameters and plasma levels of aldosterone, urea and PTH. In addition, the restoration of vitamin D levels reestablished the amount of MCP1 and the renal expressions of CD68+ and CD3+ cells in the VDD+IRI+R rats. Also, VDD+IRI+R rats showed a restoration regarding the amount of collagen type III and renal expressions of fibronectin, vimentin and α-SMA. Such changes were also accompanied by a reestablishment on the renal expression of VDR, Klotho, JG12, and TGF-β1. Our findings indicate that the restoration of vitamin D levels not only improved the renal function and hemodynamics but also reduced the inflammation and fibrosis lesions observed in I/R-AKI associated with VDD. Thus, monitoring of vitamin D status as well as its replacement in the early stages of kidney injury may be a therapeutic alternative in the mitigation of renal disease progression.

## Introduction

Worldwide, the prevalence of chronic kidney disease (CKD) has been increasing over the years (1, 2). Among others, diabetes, hypertension and obesity are well-known aggravating factors for CKD. On the other hand, growing evidences have been showing that *nontraditional* risk factors such as acute kidney injury (AKI) and hypovitaminosis D could also be involved in the progression of CKD (1–4). Based on that, many strategies for prevention and/or slowing the progression of renal diseases have been determined (1, 2).

In recent decades, advances in vitamin D research have been showing the importance of this hormone status and its beneficial effects in many diseases (5–7). Vitamin D [25(OH)D] is a multifunctional hormone that plays an important role in mineral homeostasis and it is also responsible for kidney protection and regulation of several physiological activities (3, 4, 7). Vitamin D is synthesized directly from the skin following UVB exposure or by diet (8–10). Once in the circulation, the first hydroxylation occurs in the liver by specific cytochrome enzymes becoming 25(OH)D_3._ The second and most important hydroxylation occurs mainly in the proximal convoluted tubule of the kidney by the 1α-hydroxylase enzyme, which converts 25(OH)D_3_ to 1,25(OH)_2_D_3_, the active form of vitamin D (8–10). This final renal conversion of vitamin D is strictly regulated by parathormone (PTH), phosphorus levels, and fibroblast growth factor 23 (FGF-23) as well (8–10). The nuclear vitamin D receptor (VDR) is required to vitamin D exerts its actions on targeting genes (8–10).

A large body of studies shows that vitamin D deficiency (VDD) is a common feature in CKD. In addition, a reduced VDR activation occurs early in CKD (5). Hypovitaminosis D is associated with impaired recovery of renal diseases and/or acceleration of renal damage (3). In 2015, we demonstrated that VDD aggravated AKI after ischemia/reperfusion (I/R) insult (11). We also showed that VDD exacerbated vascular impairment and enhanced inflammation during recovery from AKI after I/R insult (12). Additionally, we observed that VDD altered renal function and hemodynamic parameters in 5/6-nephrectomized (Nx) rats (3). Taken together, we demonstrated that VDD was considered an aggravating factor for tubulointerstitial damage, inflammatory pathways and renal fibrosis formation (RFF) in I/R or Nx models (3, 4, 11, 12).

Several lines of evidence have been supporting the role of vitamin D on renin-angiotensin-aldosterone system (RAAS) activity observed in clinical and/or experimental models (6, 13–15). Li et al. showed that a treatment using the active form of vitamin D decreased renin gene expression and hypertension in VDR knockout mouse (16). Studies using vitamin D or its analogs highlight their potent anti-inflammatory effects, suggesting that they should be considered as an adjunctive therapy in the treatment of various chronic diseases (6). Consistently, some studies have been showing that the administration of vitamin D may reduce glomerular inflammatory cell infiltrate, decreasing inflammation in CKD patients (6). In addition, vitamin D supplementation may prevent interstitial fibrosis (6). Tan et al. reported that VDR activation reduced the fibrotic lesions and decreased deposition of interstitial matrix components in mouse obstructive nephropathy model (17). Regarding vasculoprotective actions, vitamin D may mediate this effect by increasing nitric oxide production, inhibiting macrophages to foam cell formation or reducing the expression of adhesion molecules in endothelial cells (18).

It is well-described that AKI alters the renal hemodynamics, leads to tubular injury, and activates inflammatory, proliferative and cell death pathways (11, 12, 19, 20). The initial damage observed in the renal tissue after I/R injury suggests an important role in the course of AKI, as well as in the onset of the disease, which may lead to a predisposition to CKD (19, 20). As described above, VDD associated with AKI potentiated the injury and accelerated the progression of kidney disease (3, 4). On the other hand, a large number of studies indicates that vitamin D has multiple benefits beyond its traditional role on mineral and bone metabolism, including renoprotective actions such as the attenuation of inflammation, glomerulosclerosis, interstitial fibrosis and suppression of RAAS activity (3, 4, 6, 11, 18). Based on that, our aim was to evaluate the effect of the restoration of vitamin D levels in the progression of ischemic renal injury in rats previously deficient in vitamin D.

## Materials and Methods

### Experimental Protocol

Male Wistar rats (*Rattus novergicus*), weighting 180–200 g, were provided by the University of São Paulo, Institute of Biomedical Sciences animal facility. During the 120-day protocol, rats received vitamin D-free or standard (10,000 UI – Vitamin D_3_/kg) diets (MP Biomedicals, Irvine, CA) and had free access to tap water. All experiments followed our institutional guidelines and were approved by the local Research Ethics Committee (CEUA, registration 1023/2018).

On day 30, rats from all groups were anesthetized with 2,2,2-tribromoethanol [250 mg/kg body weight (BW)]. After that, a suprapubic incision was made for induction of ischemia/reperfusion (I/R) injury by clamping both renal arteries for 45 min. Rats were divided into three groups: (IRI) Ischemia/Reperfusion Injury (*n* = 7), fed the standard diet for 120 days; (VDD+IRI) Vitamin D Deficiency plus Ischemia/Reperfusion Injury (*n* = 8), fed the vitamin D-free diet for 120 days; and (VDD+IRI+R) Vitamin D Deficiency plus Ischemia/Reperfusion Injury plus Vitamin D Replacement (*n* = 11), fed the vitamin D-free diet in the first 30 days and just after I/R insult, on day 31, we reintroduced the standard diet in the last 90 days.

### Analysis of Urine Samples

Before the clearance studies, all rats were placed in individual metabolic cages, on a 12/12-h light/dark cycle, with free access to drinking water. We collected 24-h urine samples, centrifuged them to remove suspended material and analyzed the supernatants. We assessed urine output and urinary protein excretion was measured by a colorimetric system using a commercial kit (Labtest Diagnóstica, Minas Gerais, Brazil).

### Inulin Clearance and Hemodynamic Studies

On day 120, we anesthetized the animals with sodium thiopental (50 mg/kg BW) and then we cannulated the trachea with a PE-240 catheter for spontaneous breathing. The jugular vein was cannulated with PE-60 catheter for infusion of inulin and fluids. To monitor mean arterial pressure (MAP) and collect blood samples, the right femoral artery was catheterized with a PE-50 catheter. We assessed MAP with a data acquisition system (MP100; Biopac Systems, Santa Barbara, CA). To collect urine samples, we cannulated the bladder with a PE-240 catheter by suprapubic incision. After the surgical procedure, a loading dose of inulin (100 mg/kg BW diluted in 1 mL of 0.9% saline) was administered through the jugular vein. A constant infusion of inulin (10 mg/kg BW) was started and continued at 0.04 mL/min throughout the whole experiment. We collected three urine samples at 30-min intervals. Blood samples were obtained at the beginning and at the end of the experiment. Inulin clearance values represent the mean of three periods. Plasma and urinary inulin were determined by the anthrone method, and the glomerular filtration rate (GFR) data are expressed as mL/min/100 g BW. To measure renal blood flow (RBF), we made a median incision and dissected the left renal pedicle for isolating the renal artery. An ultrasonic flow probe was placed around the exposed renal artery, and RBF was measured (mL/min) with an ultrasonic flow meter (T402; Transonic Systems, Bethesda, MD). We divided blood pressure by RBF to calculate renal vascular resistance [(RVR), mmHg/mL/min].

### Biochemical Parameters

To evaluate plasma levels of 25(OH)D, PTH, FGF-23, aldosterone, phosphate (P_P_), calcium (P_Ca_), and urea (P_Ur_), we collected blood samples after the clearance studies. We assessed 25(OH)D, PTH, FGF-23, and aldosterone by enzyme-linked immunosorbent assay (ELISA) using commercial kits: 25-hydroxyvitamin D (ALPCO, Salem, NH); Rat Intact PTH and Mouse/Rat Intact FGF-23 (Immutopics, Inc., San Clemente, CA); and Aldosterone (Enzo Life Sciences, Farmingdale, NY). We measured P_Ca_, P_P_, and P_Ur_ by colorimetric assay (Labtest, Minas Gerais, Brazil).

### Tissue Samples Preparation

After blood samples collection, we perfused the kidneys with phosphate-buffered solution (PBS, pH 7.4). We froze the right kidneys in liquid nitrogen and stored at −80°C for Western blotting, ELISA and real-time quantitative polymerase chain reaction (qPCR). The left kidneys were removed and a fragment of the renal tissue was fixed in methacarn solution (60% methanol, 30% chloroform, 10% glacial acetic acid) for 24 h and in 70% alcohol thereafter. The kidney blocks were embedded in paraffin and cut into 4-μm sections for histological and immunohistochemical studies.

### Total Protein Isolation

Kidney samples were homogenized in ice-cold isolation solution (200 mM mannitol, 80 nM HEPES and 41 mM KOH, pH 7.5) containing a protease inhibitor cocktail (Sigma Chemical Company, St. Louis, MO) with a homogenizer (Tissue Master TM125, Omni International, Kennesaw, GA). Homogenates were centrifuged at 4,000× rpm for 30 min at 4°C to remove nuclei and cell debris. The supernatants were isolated, and protein was quantified by Bradford assay (Bio-Rad Laboratories, Hercules, CA).

### Western Blot Assay

For Western blot analysis, 100 μg of total kidney protein were separated on SDS-polyacrylamide minigels by electrophoresis (21). After transfer by electroelution to PVDF membranes (GE Healthcare Limited, Little Chalfont, UK), blots were blocked for 1 h with 5% non-fat milk in Tris-buffered saline solution. Blots were then incubated overnight with primary antibodies for anti-VDR and anti-Klotho (1:500 for both; Santa Cruz Biotechnology, Santa Cruz, CA). The labeling was visualized with a horseradish peroxidase-conjugated secondary antibody (anti-rabbit, 1:2,000, or anti-goat, 1:10,000; Sigma Chemical, St. Louis, MO) and enhanced chemiluminescence (ECL) detection (GE Healthcare Limited, Little Chalfont, UK). Kidney protein levels were further analyzed with a gel documentation system (Alliance 4.2; Uvitec, Cambridge, UK) and the software Image J for *Windows* (Image J-NIH Image). We used densitometry to quantitatively analyze the protein levels, normalizing the bands to β-actin expression (anti-β-actin, Sigma Chemical, St. Louis, MO).

### ELISA in Renal Tissue

We assessed Collagen Type III (COL3) and MCP1/CCL2 (*Monocyte Chemotactic Protein 1*) in renal tissue by ELISA using commercial kits (MyBiosource, San Diego, CA and LifeSpain BioSciences, Seattle, DC, respectively). The detection system and the quantification followed the protocols described by the manufacturers. The absorbances were obtained using the Epoch/2 device (Biotek Instruments, Winooski, VE).

### Light Microscopy

Four-micrometer histological sections of kidney tissue were stained with Masson's trichrome and examined under light microscopy. We quantified the fractional interstitial area (FIA) by analyzing tubulointerstitial involvement and glomerular tuft area as well. For histomorphometry, the images obtained by microscopy were captured on a computer screen *via* an image analyzer software (ZEN, Carl Zeiss, Munich, Germany). For FIA evaluation, we analyzed 30–40 grid fields (0.09 mm^2^ each) per kidney cortex. The interstitial areas were demarcated manually, and the proportion of the field was determined after excluding the glomeruli. For glomerular area, we calculated the arithmetic mean after analyzing approximately 80 glomeruli of each kidney section. The glomerular tuft area (μm^2^) was circulated manually and calculated automatically by the software. To minimize bias during the morphometric analysis, the observer was blinded to the treatment groups.

### Immunohistochemical Analysis

Immunohistochemistry was performed on 4-μm-thick paraffinized kidney sections mounted on 2% silane-coated glass slides. We used the following antibodies: (1:100) mouse monoclonal to CD68 (ED1; BioRad, Hercules, CA); (1:2,000) rabbit polyclonal to mannose receptor (CD206; Abcam, Cambridge, MA); (1:50) mouse monoclonal to CD3 (DAKO, Glostrup, Denmark); (1:200) mouse monoclonal to α-smooth muscle actin (α-SMA) (Millipore, Billerica, MA); (1:400) rabbit monoclonal to fibronectin (Abcam, Cambridge, MA); (1:50) mouse monoclonal to vimentin (DAKO, Glostrup, Denmark); (1:100) rabbit polyclonal to transforming growth factor-β1 (TGF-β1) (Santa Cruz Biotechnology, Santa Cruz, CA); and (1:100) mouse monoclonal to JG12, direct against to aminopeptidase P (Santa Cruz Biotechnology, Santa Cruz, CA). We subjected the kidney tissue sections to immunohistochemical (IHC) reaction according to the protocol for each primary antibody. Reaction products were detected by avidin–biotin-peroxidase (Vector Laboratories, Burlingame, CA). The color reaction was developed in 3,3-diaminobenzidine (Sigma, St. Louis, MO) and hydrogen peroxide. Counterstaining was with Harris' hematoxylin. We analyzed 30–50 renal cortex fields (0.09 mm^2^) to evaluate the immunoreactions. The volume ratios of positive areas of renal tissue (%), determined by the color limit, were obtained by an image analyzer software (ZEN, Carl Zeiss, Munich, Germany) on a computer coupled to a microscope (Axioskop 40; Carl Zeiss) and a digital camera (4, 22). To minimize bias during the IHC analysis, the observer was blinded to the treatment groups.

### Gene Expression

We performed real-time qPCR in frozen renal tissue, assessing the following genes: *VDR* (Rn00690616_m1) and *klotho* (Rn00580123_m1). We extracted and prepared total RNA by centrifugation technique using the commercial kit *SV Total RNA Isolation System* (Promega Corporation, Madison, WI). For cDNA synthesis, we used total RNA and GoTaq qPCR master mix reagent (Promega, Madison, WI). We performed real-time PCR using TaqMan (Applied Biosystems, Foster City, CA) on Step One Plus (Applied Biosystems). Primers were purchased from Applied Biosystems. We evaluated relative gene expression with the 2^−Δ*ΔCt*^ method (23), using glyceraldehyde 3-phosphate dehydrogenase (*GAPDH*) as the housekeeping gene (Rn01775763_g1).

### Statistical Analysis

All quantitative data are expressed as mean ± SEM (standard error of the mean). Differences among groups were analyzed with GraphPad Prism 5.0 software (GraphPad Software, La Jolla, CA) by one-way analysis of variance followed by the Student–Newman–Keuls test. Values of *p* < 0.05 were considered statistically significant.

## Results

### Renal Function and Hemodynamic Analysis

We did not observe any differences in BW among groups since all animals showed similar food ingestion (~25 g/day) during the 120-day protocol (data not shown). Our inulin clearance studies (mL/min/100g BW) showed that the restoration of vitamin D levels improved the renal function, evidenced by a higher GFR in the VDD+IRI+R group compared to the other groups (Table 1). Corroborating our renal function data, we found a lower plasma urea concentration (mg/dL) in the VDD+IRI+R group than in the VDD+IRI group (Table 1). In addition, we noted that the restoration of vitamin D levels reestablished MAP (mmHg), RBF (mL/min) and RVR (mmHg/mL/min) in VDD+IRI+R rats in relation to the IRI rats (Table 1). Following MAP data, we also observed a recovery regarding aldosterone plasma levels (pg/mL) in the VDD+IRI+R rats. Therefore, our data reinforce that vitamin D is a protective factor concerning renal function and hemodynamic parameters.

**Table 1:** Renal function, hemodynamic and biochemical parameters evaluated in rats after 90 days of a bilateral 45 min ischemia/reperfusion insult (IRI) on day 30.

	**IRI**	**VDD+IRI**	**VDD+IRI+R**
C_In_ (mL/min/100 g BW)	0.47 ± 0.02	0.46 ± 0.03	0.57 ± 0.02^cf^
P_Ur_ (mg/dL)	44.10 ± 2.46	54.54 ± 2.82^c^	47.45 ± 1.77^f^
MAP (mmHg)	124.9 ± 2.56	142.0 ± 3.73^a^	122.0 ± 2.56^d^
RBF (mL/min)	7.90 ± 0.43	6.08 ± 0.03^c^	8.80 ± 0.49^e^
RVR (mmHg/min)	16.01 ± 1.07	22.37 ± 0.38^a^	13.97 ± 0.85^d^
P_Aldo_ (pg/mL)	*2, 172*±328.2	*3, 612*±662.4^c^	*1, 753*±167.4^e^
Proteinuria (mg/24 h)	10.14 ± 0.64	11.99 ± 0.83	9.13 ± 0.74^f^
P_25(OH)D_ (ng/mL)	42.62 ± 2.28	<0.780^a^	42.89 ± 2.58^d^
P_PTH_ (pg/mL)	806.5 ± 89.2	*1, 378*±215.4^c^	778.4 ± 135.4^f^
P_FGF−23_ (pg/mL)	356.4 ± 37.5	566.1 ± 26.2^b^	653.0 ± 62.1^a^
FE_Ca_ (%)	0.65 ± 0.10	0.64 ± 0.12	0.54 ± 0.05
FE_P_ (%)	8.89 ± 0.90	15.97 ± 1.01^b^	12.61 ± 1.39^c^

*VDD, vitamin D deficiency; R, vitamin D replacement. Diet protocol: IRI, standard diet for 120 days; VDD+IRI, vitamin D-free diet for 120 days; and VDD+IRI+R, vitamin D-free diet in the first 30 days and standard diet in the last 90 days. C_In_, inulin clearance; BW, body weight; P_Ur_, plasma urea concentration; MAP, mean arterial pressure; RBF, renal blood flow; RVR, renal vascular resistance; P_Aldo_, plasma aldosterone concentration; P_25(OH)D_, plasma 25 hydroxyvitamin D concentration; P_PTH_, plasma parathormone concentration; P_FGF−23_, plasma fibroblast growth factor 23 concentration; FE_Ca_, fractional excretion of calcium; FE_P_, fractional excretion of phosphorus. Values are mean ± SEM. ^a^p < 0.001, ^b^p < 0.01, ^c^p < 0.05 vs. IRI; ^d^p < 0.001, ^e^p < 0.01, ^f^p < 0.05 vs. VDD+IRI*.

### Vitamin D, PTH, and Other Biochemical Parameters

Regarding vitamin D plasma levels on day 30, rats which received the standard diet presented 31.83 ± 1.60 ng/mL while those which received the vitamin D-free diet showed a lower concentration of this hormone [6.87 ± 0.38 (*p* < 0.001)]. At the end of 120 day-protocol, VDD+IRI+R rats showed reestablished plasma levels of 25(OH)D in comparison to IRI rats (Table 1). Based on the daily food ingestion, we assume that our IRI and VDD+IRI+R rats consumed ~250 UI – Vitamin D_3_/day. Concerning intact PTH (pg/mL) data, we found significant lower plasma levels of this hormone in the IRI and VDD+IRI+R rats than in the VDD+IRI rats (Table 1). Plasma levels of calcium and phosphorus did not change among the experimental groups. As also shown in Table 1, we observed a significant and lower proteinuria (mg/24 h) in the VDD+IRI+R group than in the VDD+IRI group. Neither fractional excretion of calcium (Table 1) nor urinary volume presented differences among the groups. Meanwhile, the fractional excretion of phosphorus (%) was higher in the VDD+IRI and VDD+IRI+R groups than in the IRI group (Table 1).

### Klotho-FGF-23 Axis

**Figure 1:**
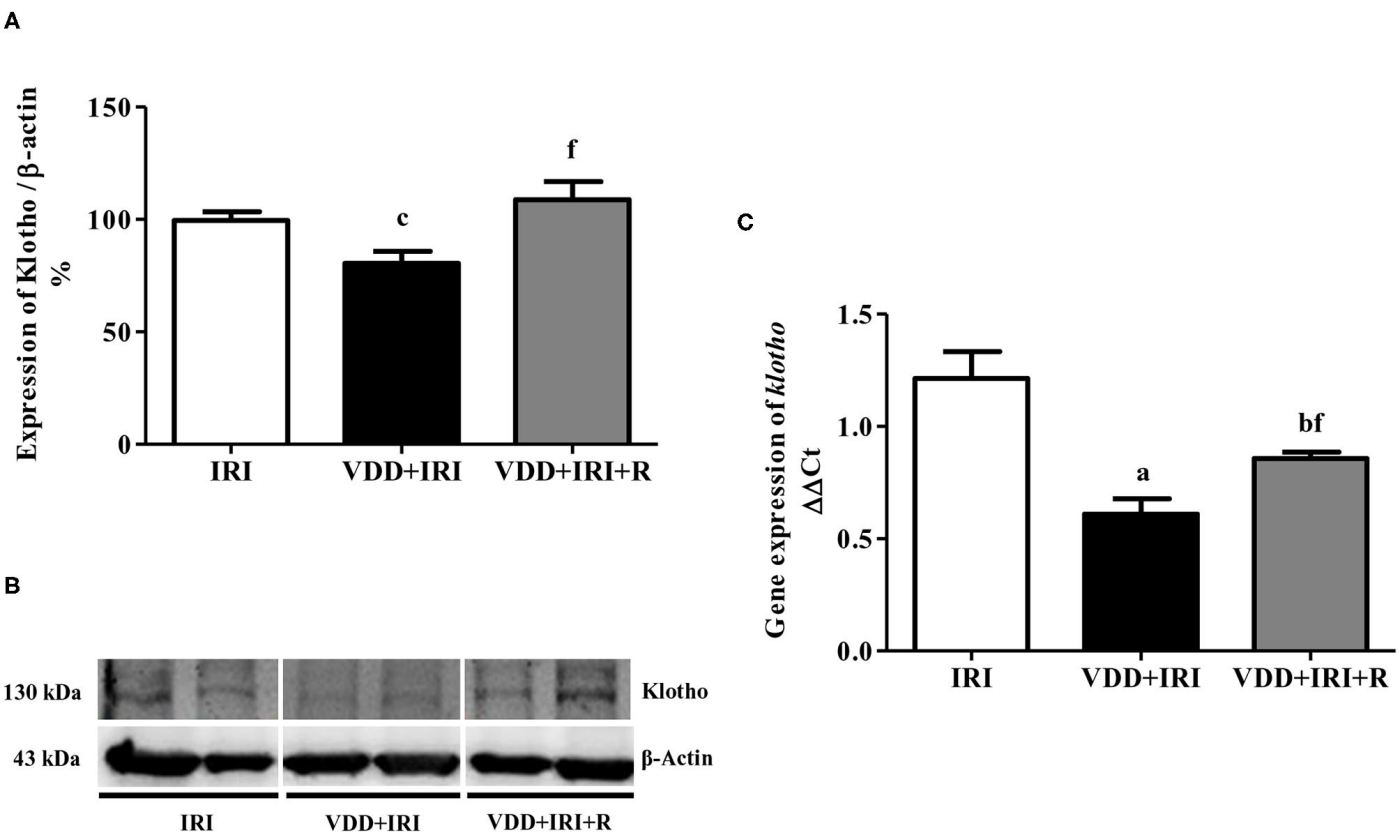
Semiquantitative immunoblotting and real-time quantitative polymerase chain reaction for renal Klotho expression evaluated 90 days after ischemia/reperfusion insult (IRI) on day 30. **(A)** Densitometric analysis of samples from IRI, VDD+IRI and VDD+IRI+R rats. **(B)** Representative immunoblots which reacted with anti-Klotho revealing a 130 kDa band. Note that vitamin D replacement restored the expression of Klotho protein in the VDD+IRI+R rats. **(C)** Bar graph of *klotho* gene expression values. Data are mean ± SEM. ^a^*p* < 0.001, ^b^*p* < 0.01, ^c^*p* < 0.05 vs. IRI; ^f^ *p* < 0.05 vs. VDD+IRI. VDD, vitamin D deficiency; R, vitamin D replacement. Diet protocol: IRI, standard diet for 120 days; VDD+IRI, vitamin D-free diet for 120 days; and VDD+IRI+R, vitamin D-free diet in the first 30 days and standard diet in the last 90 days.

During kidney disease progression, it is usually observed a reduction in Klotho expression following alterations in FGF-23 (24). We noted a significant higher plasma levels of FGF-23 in the VDD+IRI and VDD+IRI+R groups than in the IRI group (Table 1). In addition, we found a higher expression of Klotho protein (%) in the VDD+IRI+R rats in comparison to the VDD+IRI rats (Figure 1). Corroborating our Western Blot data, we found a similar profile concerning gene expression of *klotho* (Figure 1). Thus, our results showed that sufficient vitamin D levels contributed to keep higher levels of Klotho and FGF-23, which could be involved in slowing the progression of CKD.

### Histomorphological Studies

**Figure 2:**
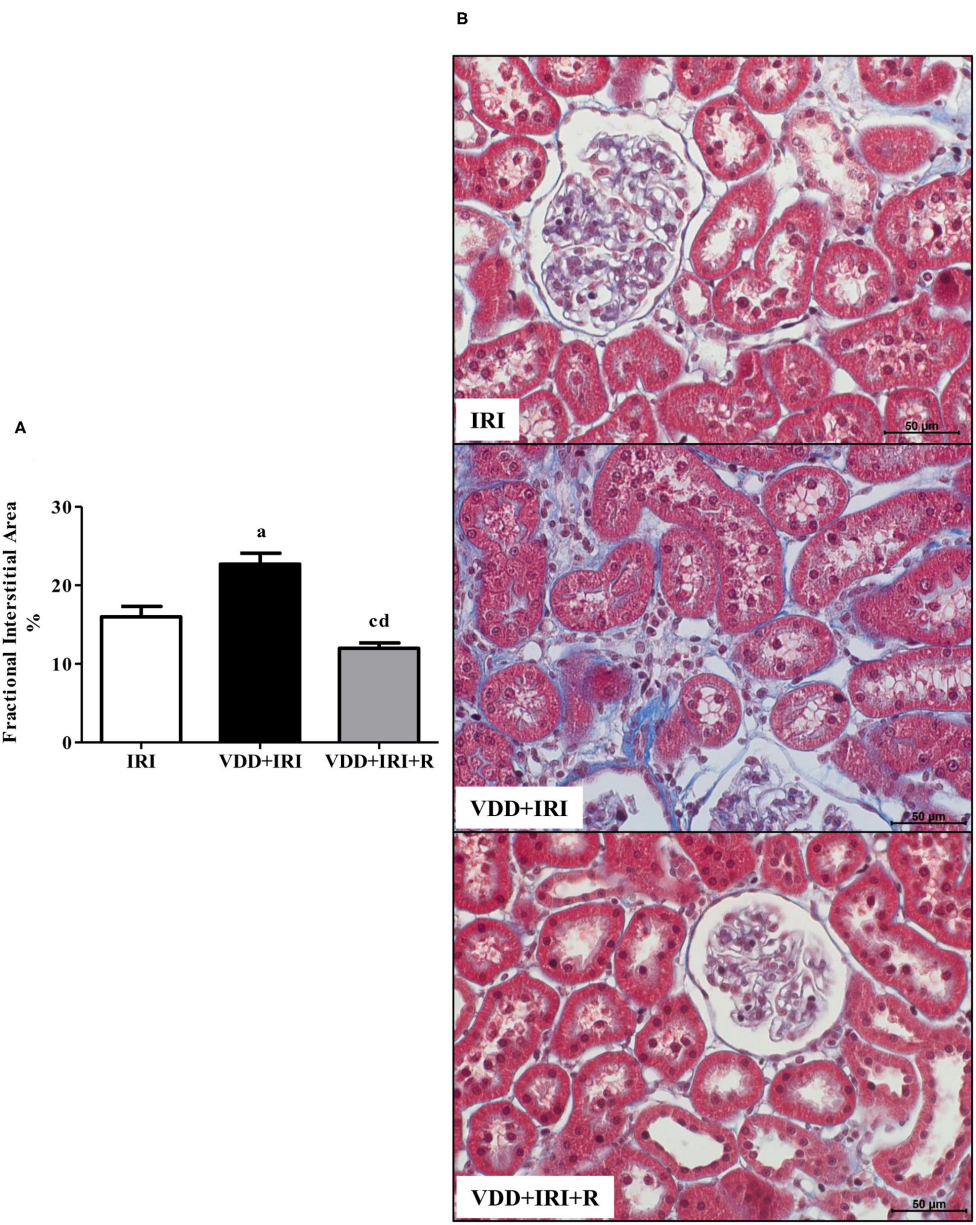
Fractional interstitial area (FIA) in the renal cortex evaluated 90 days after ischemia/reperfusion insult (IRI) on day 30. **(A)** Bar graph of FIA values. **(B)** Representative photomicrographs of renal histological changes from a IRI, VDD+IRI and VDD+IRI+R rat (×400). Note that FIA is significantly smaller in VDD+IRI+R than in the other groups. Data are mean ± SEM. ^a^*p* < 0.001, ^c^*p* < 0.05 vs. IRI; ^d^*p* < 0.001 vs. VDD+IRI. VDD, vitamin D deficiency; R, vitamin D replacement. Diet protocol: IRI, standard diet for 120 days; VDD+IRI, vitamin D-free diet for 120 days; and VDD+IRI+R, vitamin D-free diet in the first 30 days and standard diet in the last 90 days.

We performed morphometric studies to evaluate the FIA of the renal cortex. We observed more evident alterations in the tubulointerstitial compartment, featuring increased interstitial expansion, renal fibrosis, and inflammatory cell infiltrate in the VDD+IRI rats. The restoration of vitamin D levels attenuated the morphological alterations in the renal cortex from VDD+IRI+R rats not only in comparison to the VDD+IRI rats but also to the IRI rats (Figure 2). Based on our results, vitamin D exerted an important role on interstitial morphological alterations observed in our experimental model.

### Vitamin D Replacement and Inflammation

**Figure 3:**
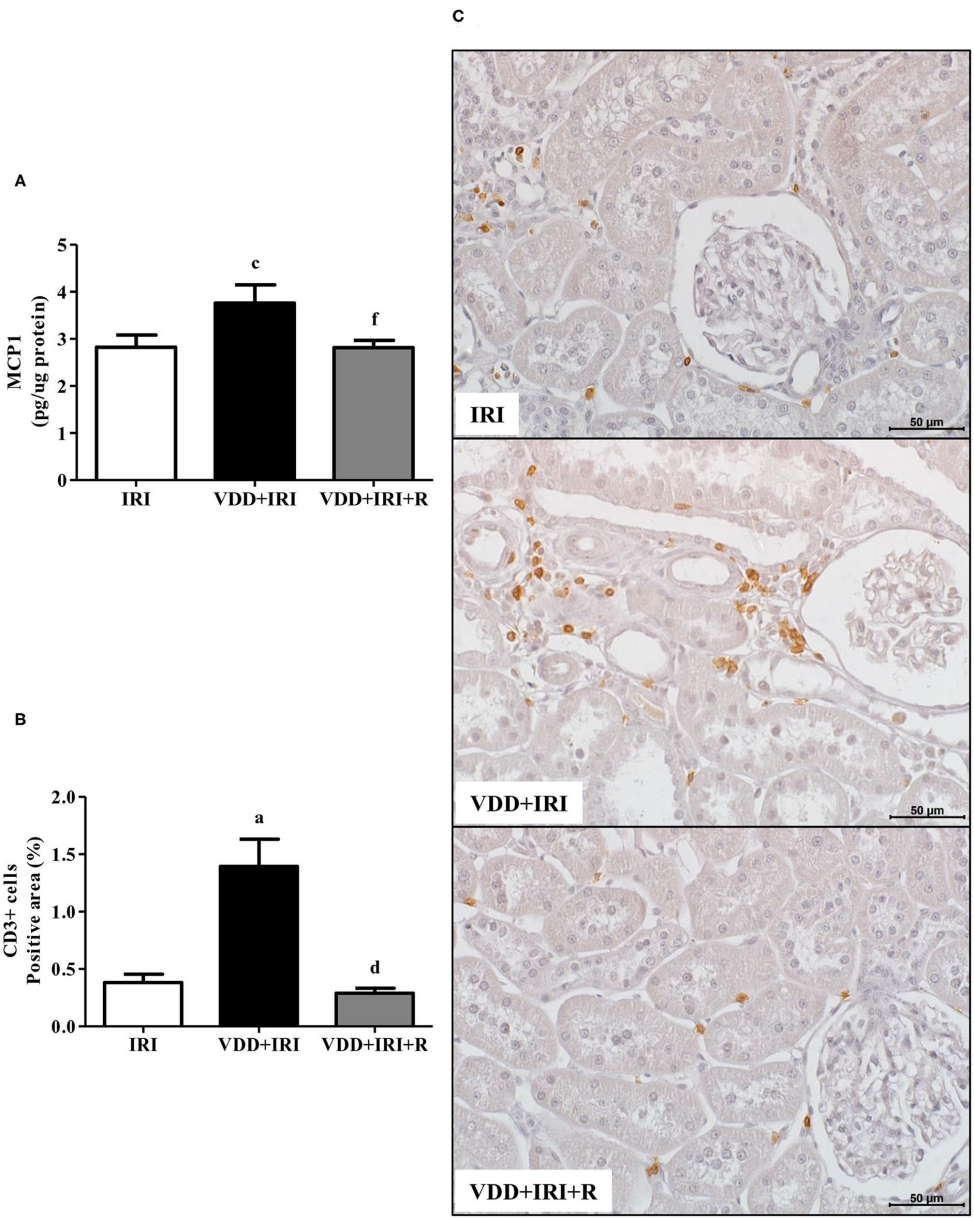
Quantitative amount of *Monocyte Chemotactic Protein 1* (MCP1)—ELISA and immunohistochemical analysis of CD3+ cells (T cells) expression in the kidney tissue evaluated 90 days after ischemia/reperfusion insult (IRI) on day 30. **(A)** Bar graphs of MCP1 and **(B)** CD3+ cells expression values. **(C)** Representative photomicrographs of immunostaining for CD3+ cells in the renal cortex from an IRI, VDD+IRI and VDD+IRI+R rat (×400). Note that vitamin D replacement restored the expression of both MCP1 and CD3+ cells in the VDD+IRI+R rats. Data are mean ± SEM. ^a^*p* < 0.001, ^c^*p* < 0.05 vs. IRI; ^d^*p* < 0.001, ^f^*p* < 0.05 vs. VDD+IRI. VDD, vitamin D deficiency; R, vitamin D replacement. Diet protocol: IRI, standard diet for 120 days; VDD+IRI, vitamin D-free diet for 120 days; and VDD+IRI+R, vitamin D-free diet in the first 30 days and standard diet in the last 90 days.

**Figure 4:**
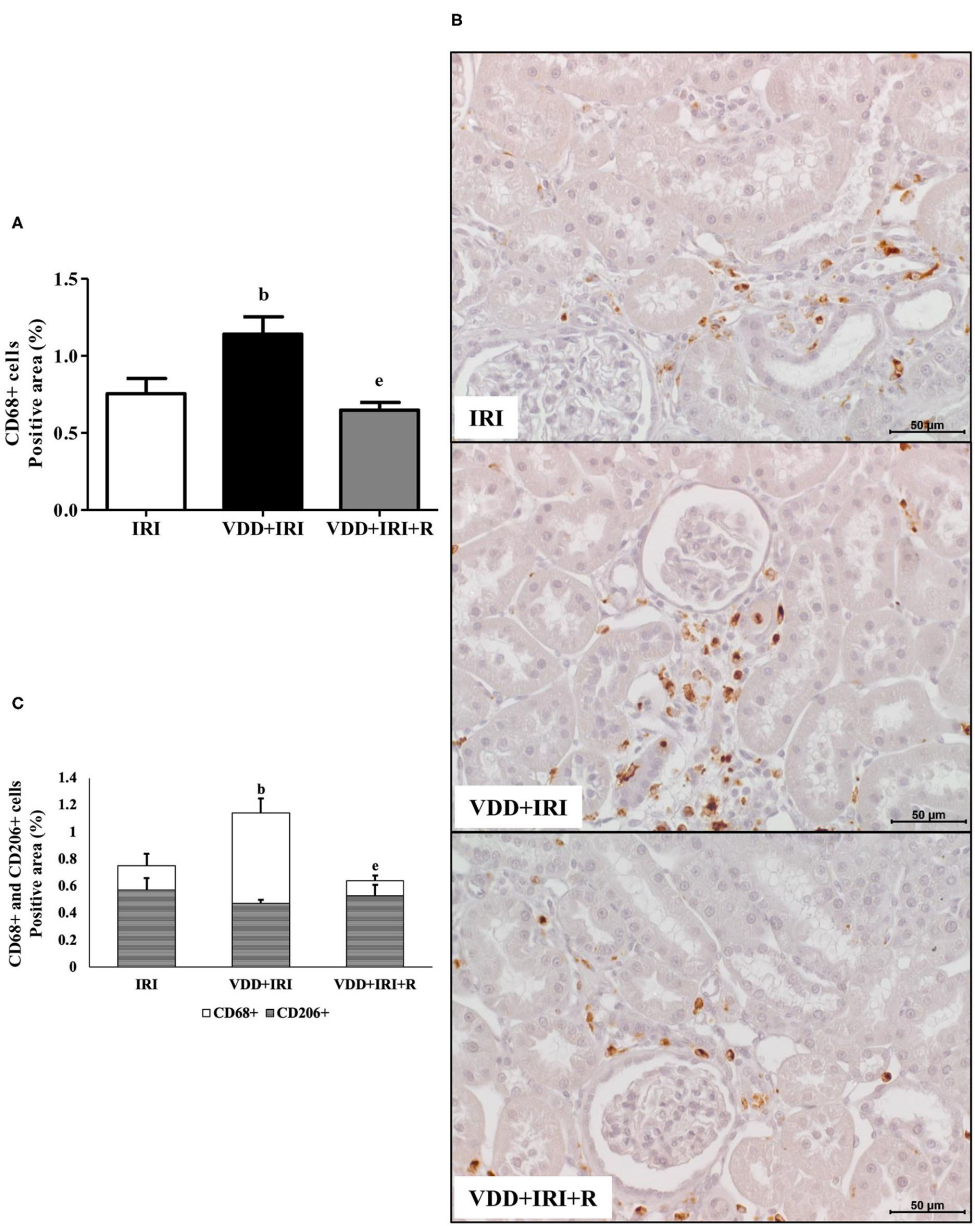
Immunohistochemical analysis of CD68+ cells (M1+M2 macrophages) and CD206+ cells (M2 macrophages) expression in the kidney tissue evaluated 90 days after ischemia/reperfusion insult (IRI) on day 30. **(A)** Bar graph of CD68+ cells expression values. **(B)** Representative photomicrographs of immunostaining for CD68+ cells in the renal cortex from an IRI, VDD+IRI and VDD+IRI+R rat (×400). Note that vitamin D replacement restored the expression of CD68+ cells in the VDD+IRI+R rats. **(C)** Bar graph regarding the proportion of CD206+ cells in relation to the amount of CD68+ cells. Data are mean ± SEM. ^b^*p* < 0.01, vs. IRI; ^e^*p* < 0.01 vs. VDD+IRI. VDD, vitamin D deficiency; R, vitamin D replacement. Diet protocol: IRI, standard diet for 120 days; VDD+IRI, vitamin D-free diet for 120 days; and VDD+IRI+R, vitamin D-free diet in the first 30 days and standard diet in the last 90 days.

We assessed the renal amount of *MCP1* (pg/μg protein) by ELISA as well as the renal expression of CD3+ (T cells) and CD68+ (macrophages) cells by IHC studies (%). As shown in Figure 3, we found a higher *MCP1* amount in the VDD+IRI rats than in the IRI and VDD+IRI+R rats. Likewise, Figure 3 shows a higher renal expression of CD3+ cells in the VDD+IRI group than in both IRI and VDD+IRI+R groups. By using an anti-CD68 antibody, we immunolocalized the macrophage population as a whole (M1+M2) in the renal cortex. As illustrated in Figure 4, the percentage of CD68+ cells in the tubulointerstitial compartment was higher in the VDD+IRI rats than in the IRI and VDD+IRI+R rats. In addition, we evaluated the proportion of CD206+ cells (M2 macrophages) in relation to the whole amount of macrophages stained with CD68 (M1+M2 macrophages). CD206 is also known as mannose receptor, which is an exclusive marker for M2 macrophages (3, 25). Although without a significant difference, we observed an upward trend in the expression of CD206+ cells in the VDD+IRI+R group in comparison to the VDD+IRI group (Figure 4). Therefore, our results strengthen the important role of vitamin D on the modulation of renal inflammation.

### VDR Expression and Renal Fibrosis Formation

**Figure 5:**
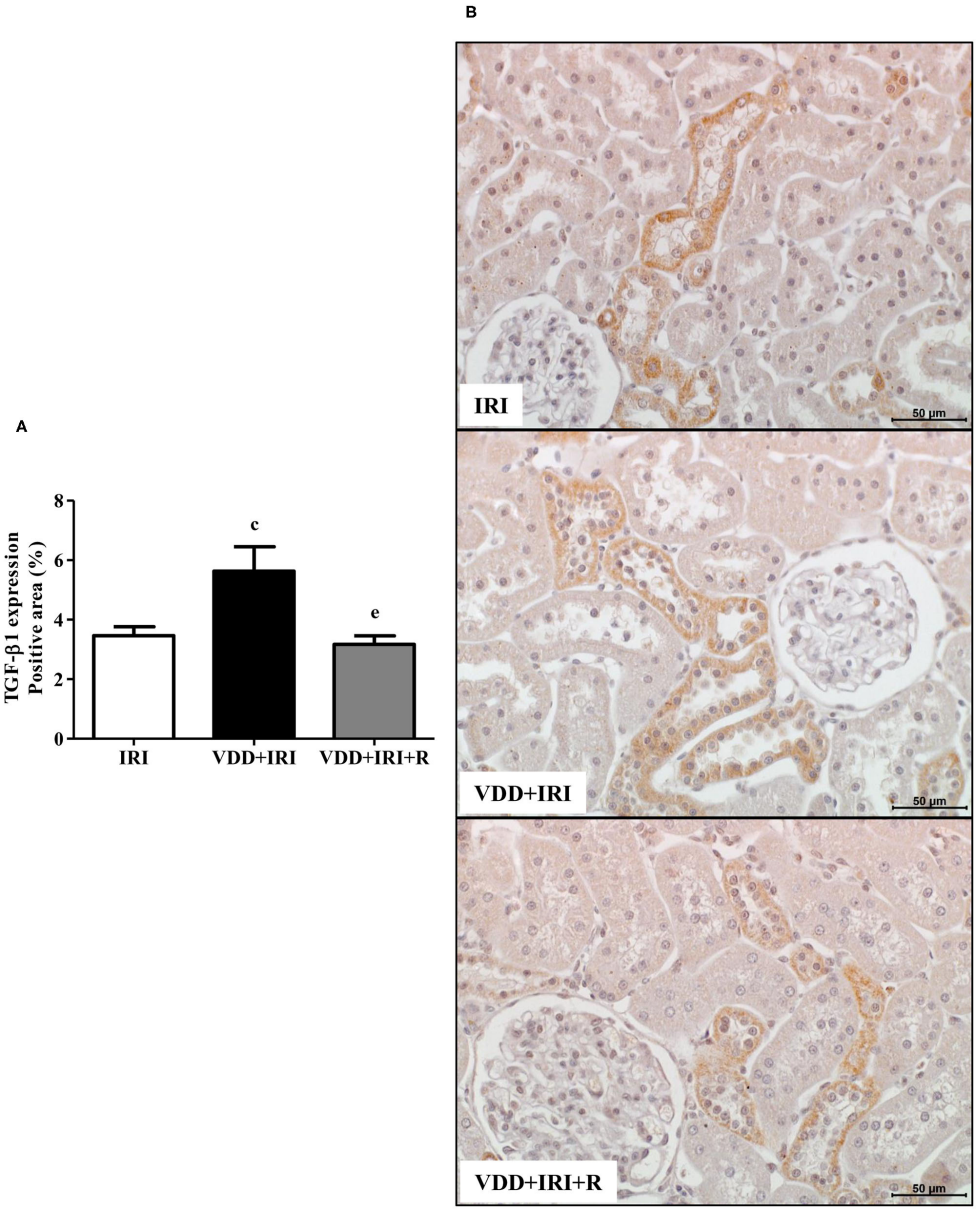
Immunohistochemical analysis for transforming growth factor-β1 (TGF-β1) expression in the kidney tissue. **(A)** Bar graph of TGF-β1 values. **(B)** Representative photomicrographs of immunostaining for TGF-β1 in the renal cortex from an IRI, VDD+IRI and VDD+IRI+R rat (×400). Note that vitamin D replacement restored the expression of TGF-β1 in the VDD+IRI+R rats. Data are mean ± SEM. ^c^*p* < 0.05, vs. IRI; ^e^*p* < 0.01 vs. VDD+IRI. VDD, vitamin D deficiency; R, vitamin D replacement. Diet protocol: IRI, standard diet for 120 days; VDD+IRI, vitamin D-free diet for 120 days; and VDD+IRI+R, vitamin D-free diet in the first 30 days and standard diet in the last 90 days.

**Figure 6:**
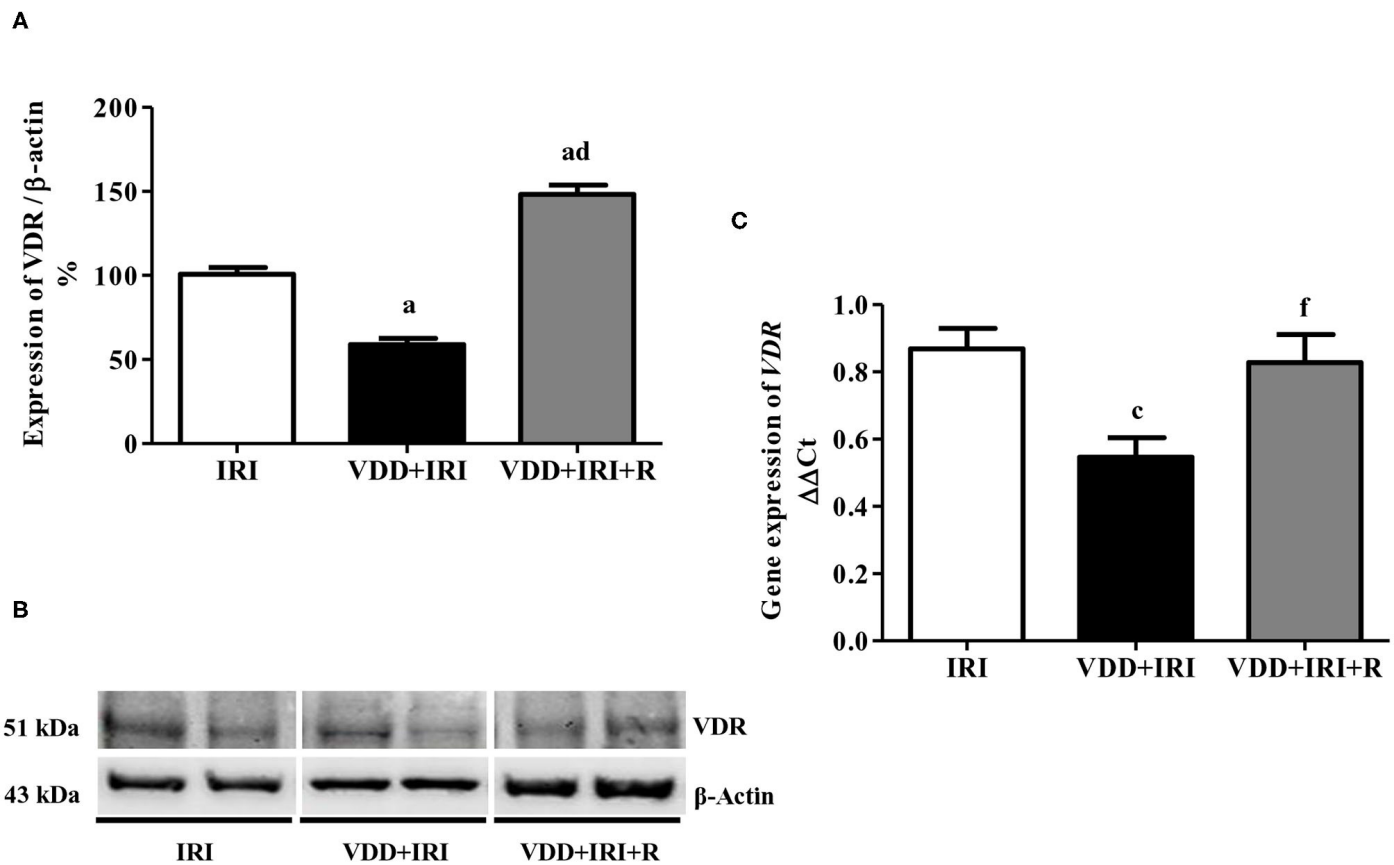
Semiquantitative immunoblotting and real-time quantitative polymerase chain reaction for renal VDR expression evaluated 90 days after ischemia/reperfusion insult (IRI) on day 30. **(A)** Densitometric analysis of samples from IRI, VDD+IRI and VDD+IRI+R rats. **(B)** Representative immunoblots which reacted with anti-VDR revealing a 51 kDa band. **(C)** Bar graph of *VDR* gene expression values. Note that vitamin D replacement restored the expression of VDR in the VDD+IRI+R rats. Data are mean ± SEM. ^a^*p* < 0.001, ^c^*p* < 0.05 vs. IRI; ^d^*p* < 0.001, ^f^*p* < 0.05 vs. VDD+IRI. VDD, vitamin D deficiency; R, vitamin D replacement. Diet protocol: IRI, standard diet for 120 days; VDD+IRI, vitamin D-free diet for 120 days; and VDD+IRI+R, vitamin D-free diet in the first 30 days and standard diet in the last 90 days.

TGF-β is the most important pro-fibrotic cytokine and vitamin D is a hormone that participates in several tissue and cellular processes involved in the suppression of renal fibrosis formation (RFF) (6, 26). Therefore, we analyzed the renal expression of TGF-β1 and VDR in all groups. We observed a higher expression of TGF-β1 (%) in the VDD+IRI group than in the IRI and VDD+IRI+R groups (Figure 5). Concerning VDR (%), we found an impressive higher expression of this receptor in the VDD+IRI+R group than in the other groups (Figure 6). As expected, VDR expression was significantly lower in the VDD+IRI rats than in the IRI rats (Figure 6). In addition, *VDR* gene expression (ΔΔCt) was lower in the VDD+IRI rats than in the other groups (Figure 6). Thus, this set of data indicated that the restoration of vitamin D levels reestablished the TGF-β1 expression, increased the VDR amount, and attenuated the morphological alterations observed in the kidneys from VDD+IRI+R rats. Of note, these findings reinforce the importance of maintaining adequate levels of vitamin D, which could contribute to the modulation of the TGF-β signaling cascade and RFF.

### Extracellular Matrix Components

**Figure 7:**
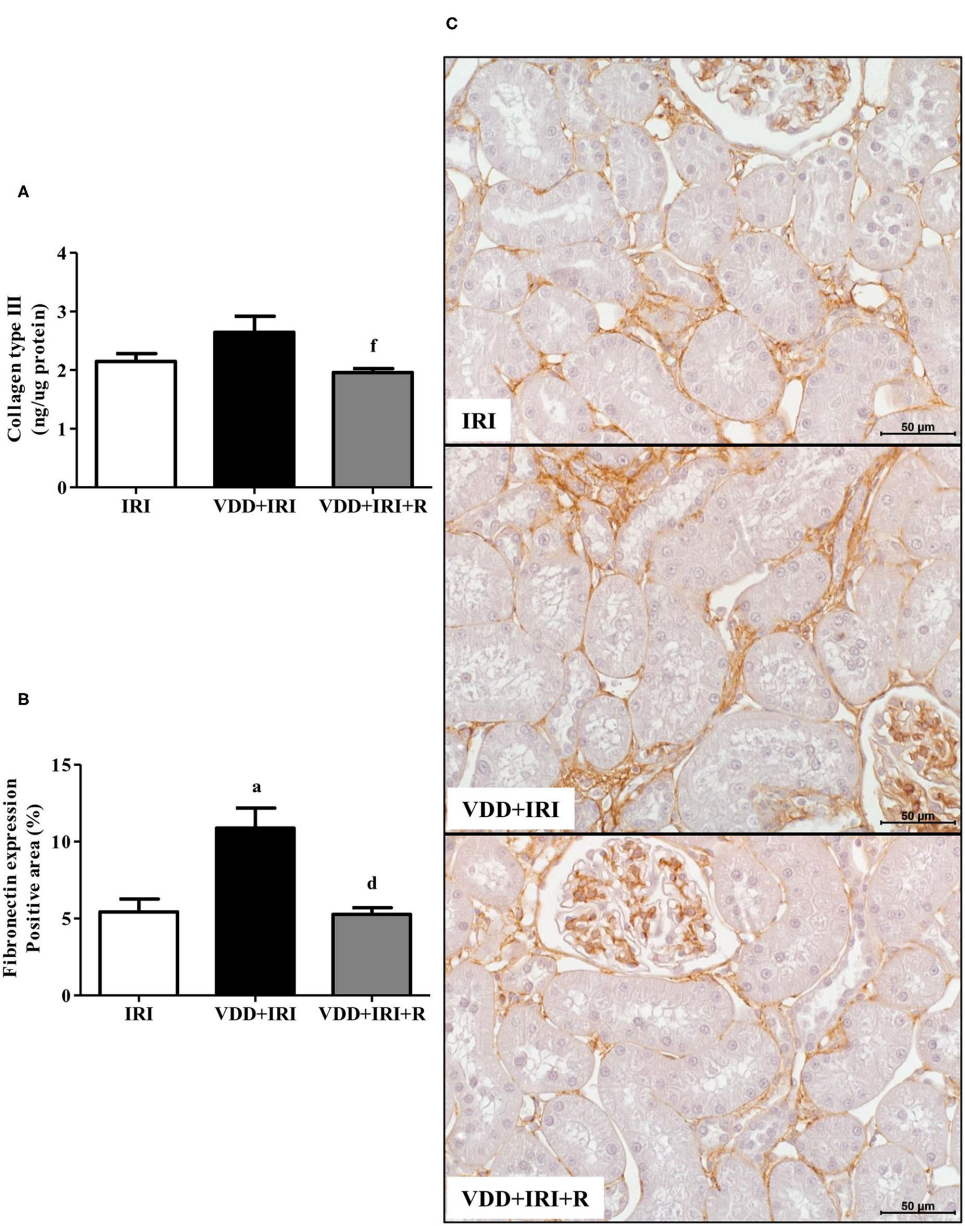
Quantitative amount of collagen type III (COL3)—ELISA and immunohistochemical analysis for fibronectin expression in the kidney tissue evaluated 90 days after ischemia/reperfusion insult (IRI) on day 30. **(A)** Bar graphs of COL3 and **(B)** fibronectin expression values. **(C)** Representative photomicrographs of immunostaining for fibronectin in the renal cortex from an IRI, VDD+IRI and VDD+IRI+R rat (×400). Note that vitamin D replacement restored the expression of both COL3 and fibronectin in the VDD+IRI+R rats. Data are mean ± SEM. ^a^*p* < 0.001 vs. IRI; ^d^*p* < 0.001, ^f^*p* < 0.05 vs. VDD+IRI. VDD, vitamin D deficiency; R, vitamin D replacement. Diet protocol: IRI, standard diet for 120 days; VDD+IRI, vitamin D-free diet for 120 days; and VDD+IRI+R, vitamin D-free diet in the first 30 days and standard diet in the last 90 days.

As mentioned in morphometric studies, our results showed that the restoration of vitamin D levels attenuated the tubulointerstitial damage and RFF as well. This process is characterized by the production and secretion of many extracellular matrix (ECM) components (27). Therefore, we performed ELISA for COL3 and immunostaining for fibronectin, both fibrous components of ECM. Figure 7 shows that the COL3 amount (ng/μg protein) was lower in the VDD+IRI+R rats than in the VDD+IRI rats. The same figure displays that the renal fibronectin expression (%) was higher in the VDD+IRI rats than in the IRI and VDD+IRI+R rats (Figure 7). Noteworthy, these findings demonstrate that the restoration of vitamin D levels was able to reduce the expression of ECM markers in the kidneys from VDD+IRI+R rats in comparison to the VDD+IRI rats.

### Phenotypic Alteration of Renal Cells

**Figure 8:**
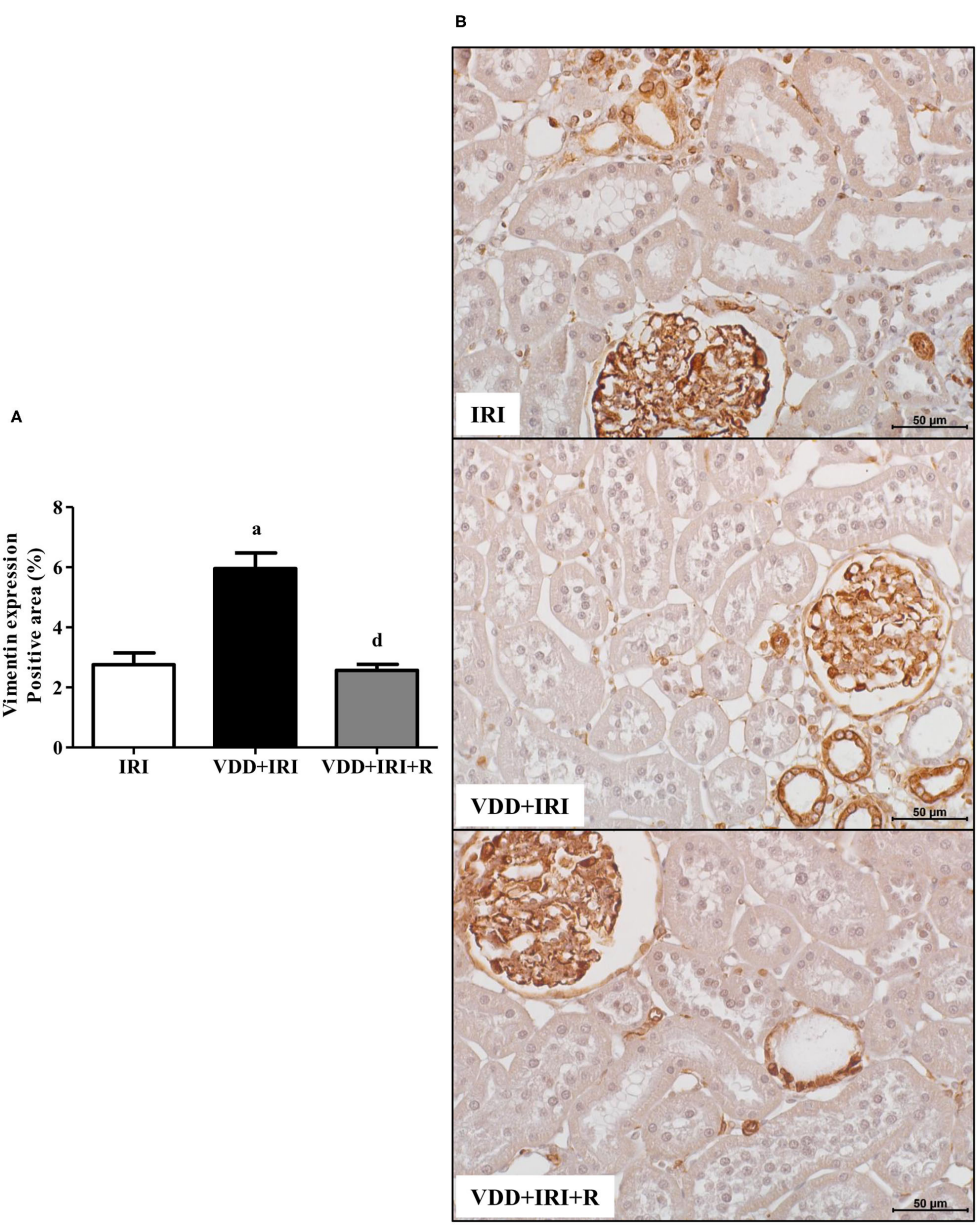
Immunohistochemical analysis for vimentin expression in the kidney tissue evaluated 90 days after ischemia/reperfusion insult (IRI) on day 30. **(A)** Bar graph of vimentin expression values. **(B)** Representative photomicrographs of immunostaining for vimentin in the renal cortex from an IRI, VDD+IRI and VDD+IRI+R rat (×400). Note that vitamin D replacement restored the expression of vimentin in the VDD+IRI+R rats. Data are mean ± SEM. ^a^ *p* < 0.001, vs. IRI; ^d^ *p* < 0.001 vs. VDD+IRI. VDD: vitamin D deficiency; R: vitamin D replacement. Diet protocol: IRI, standard diet for 120 days; VDD+IRI, vitamin D-free diet for 120 days; and VDD+IRI+R, vitamin D-free diet in the first 30 days and standard diet in the last 90 days.

**Figure 9:**
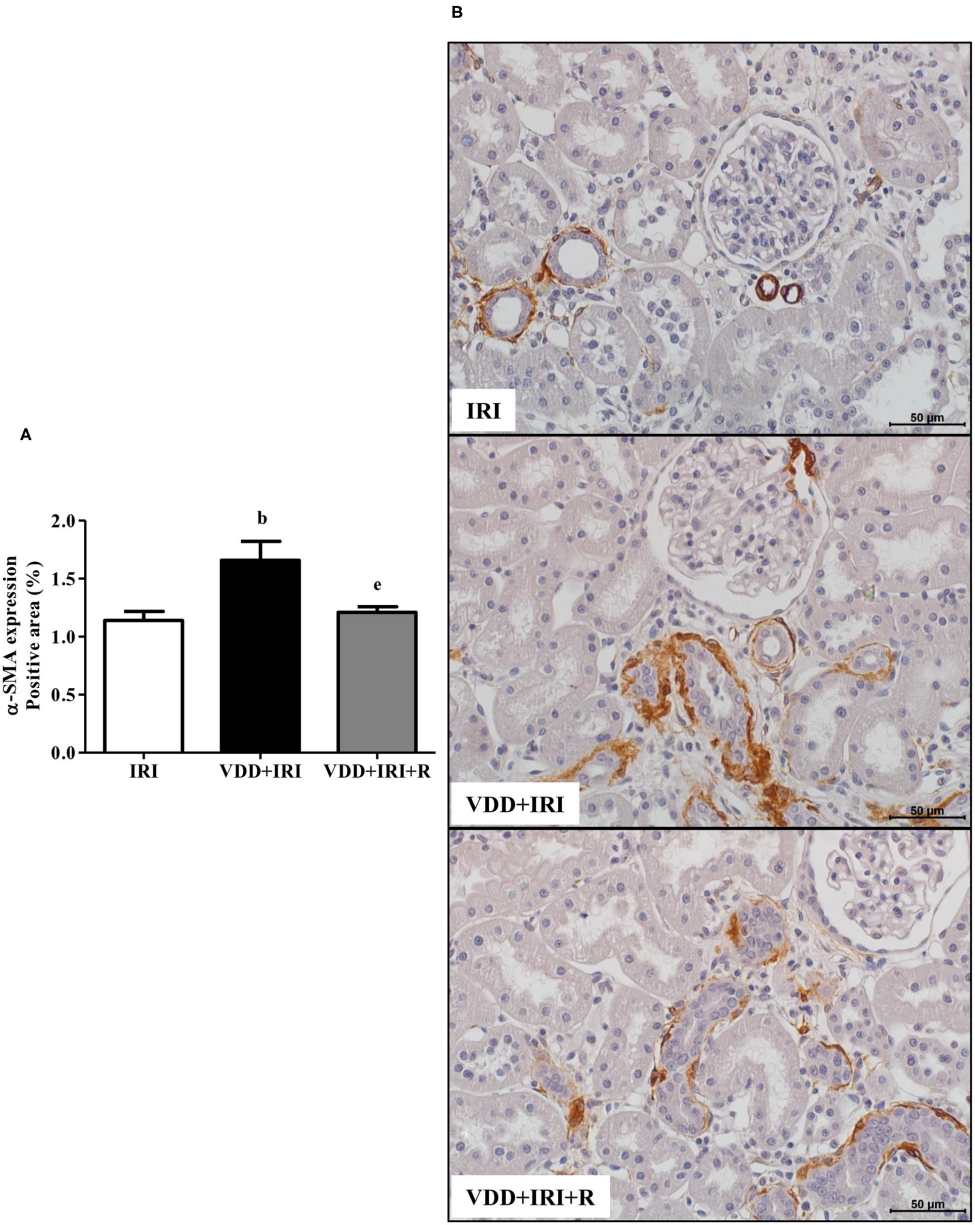
Immunohistochemical analysis for α-smooth muscle actin (α-SMA) expression in the kidney tissue evaluated 90 days after ischemia/reperfusion insult (IRI) on day 30. **(A)** Bar graph of α-SMA expression values. **(B)** Representative photomicrographs of immunostaining for α-SMA in the renal cortex from an IRI, VDD+IRI and VDD+IRI+R rat (×400). Note that vitamin D replacement restored the expression of α-SMA in the VDD+IRI+R rats. Data are mean ± SEM. ^b^*p* < 0.01, vs. IRI; ^e^*p* < 0.01 vs. VDD+IRI. VDD, vitamin D deficiency; R, vitamin D replacement. Diet protocol: IRI, standard diet for 120 days; VDD+IRI, vitamin D-free diet for 120 days; and VDD+IRI+R, vitamin D-free diet in the first 30 days and standard diet in the last 90 days.

In addition to fibrous components of ECM, we also evaluated the presence of phenotypic alteration of renal tubular cells. We used vimentin antibody to detect tubular injury and expression of α-SMA for interstitial fibroblast activation. The percentage of positive area (%) for both markers was higher in the VDD+IRI rats than in the other groups (Figures 8 and 9). Of note, vitamin D replacement restored the expression of vimentin and α-SMA in the VDD+IRI+R rats in comparison to the IRI rats, showing the role of this hormone on epithelial–mesenchymal transition (EMT).

### Effects of Vitamin D Replacement on Glomerular Vascular Endothelium

**Figure 10:**
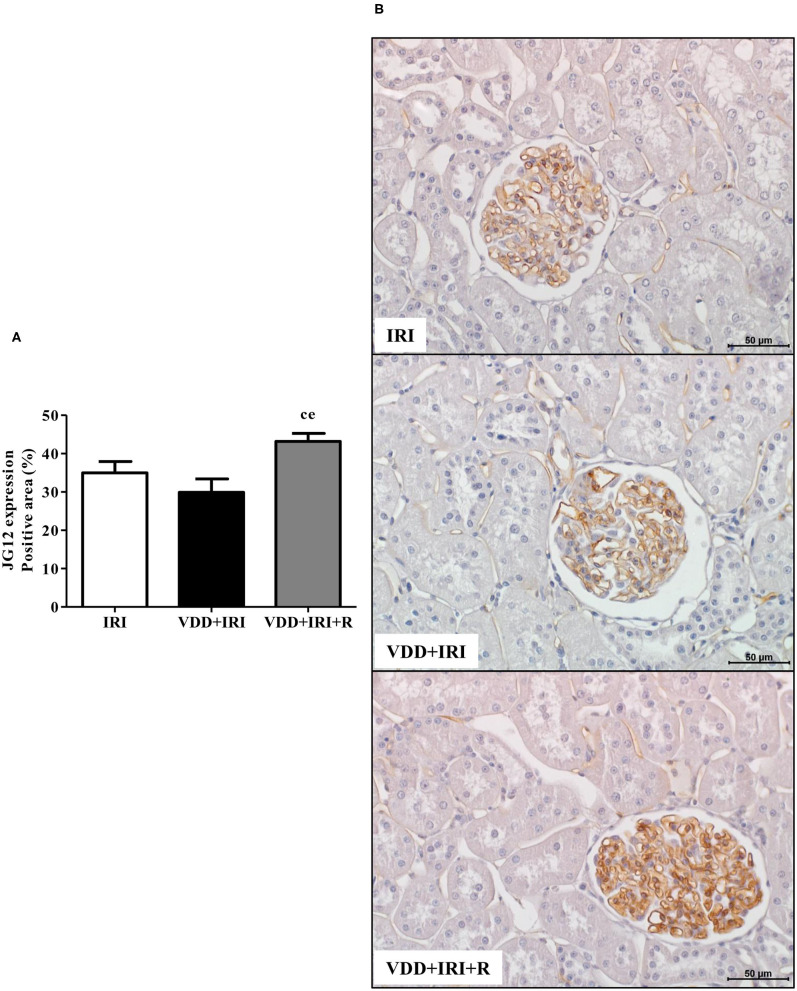
Immunohistochemical analysis for aminopeptidase P (JG12) expression in the kidney tissue evaluated 90 days after ischemia/reperfusion insult (IRI) on day 30. **(A)** Bar graph of JG12 expression values. **(B)** Representative photomicrographs of immunostaining for JG12 in the renal cortex from an IRI, VDD+IRI and VDD+IRI+R rat (×400). Note that the restauration of vitamin D levels ameliorated the expression of JG12 in the VDD+IRI+R rats. Data are mean ± SEM. ^c^*p* < 0.05, vs. IRI; ^e^*p* < 0.01 vs. VDD+IRI. VDD, vitamin D deficiency; R, vitamin D replacement. Diet protocol: IRI, standard diet for 120 days; VDD+IRI, vitamin D-free diet for 120 days; and VDD+IRI+R, vitamin D-free diet in the first 30 days and standard diet in the last 90 days.

We also evaluated whether the restoration of vitamin D levels could contribute toward improving the integrity of the vascular endothelium in the progression of CKD. We performed IHC studies to assess the expression of JG12, a specific marker for the vascular endothelium. Within the glomerular capsule, JG12 is only expressed on the surface of the capillary endothelium (3). As shown in Figure 10, JG12 staining per glomerular tuft area (%) was significantly higher in the VDD+IRI+R rats than in the other groups. Hence, the restoration of vitamin D levels ameliorated the integrity of glomerular vascular endothelium in the VDD+IRI+R rats, reinforcing its contribution on the improvement of renal function previously described.

## Discussion

In the present study, we observed that the restoration of vitamin D levels after I/R injury in rats previously deficient in vitamin D significantly improved the functions of the kidney as a whole. VDD+IRI+R rats presented a significant improvement in GFR and a smaller FIA (fibrosis and inflammatory cell infiltrate). Furthermore, we observed a reestablishment regarding the hemodynamic parameters and plasma levels of aldosterone, urea and PTH provided by the restoration of vitamin D levels. We also demonstrated that the restoration of vitamin D levels reestablished the expression of ECM components and modulated the phenotypic alteration of renal tubular cells as well. In addition, VDD+IRI+R group presented an improvement of renal protein handling and a higher expression of JG12 on the glomerular vascular endothelium. All those changes were accompanied by a higher expression of VDR, a restored expression of Klotho and TGF-β1 and an increased plasma levels of FGF-23 observed in the VDD+IRI+R rats.

Our results showed that upon returning the standard diet to the VDD+IRI+R rats after 30 days under vitamin D-free diet, there was an effective restoration of the 25(OH)D plasma levels in these animals. As described earlier, vitamin D plasma level reflects vitamin D intake from diet and supplements, as well as skin synthesis (4, 28). Here, our VDD+IRI+R rats presented an important and significant amelioration of the renal function estimated by inulin clearance, which was accompanied by lower levels of plasma urea in that group. Previous studies from our research group demonstrated that VDD is an aggravating factor for I/R-AKI, which was associated with a progressive and significant impairment of the renal tissue (4, 11, 12). The pathogenesis of AKI involves a multifactorial process and early prevention and treatment are considered the best therapeutic options (29). In 2011, de Boer et al. suggested that hypovitaminosis D is a risk factor for CKD, showing that vitamin D supplementation slowed the progression of renal disease (30). Supporting this information, recent studies also reaffirmed that the use of vitamin D metabolites and its analogs in the treatment of CKD was helpful in terms of slowing the risk of CKD progression (31, 32). Our data show that the simple restoration of vitamin D levels by the diet was sufficient to improve the renal function of the VDD+IRI+R rats.

It has been known that hypovitaminosis D in renal I/R injury compromises the blood pressure control through alterations in RAAS, endothelium and vascular smooth cells (4, 11, 12). On the other hand, studies conducted in humans and animals show that vitamin D status can modulate the RAAS activity, mainly by lowering renin synthesis (6, 15, 33). In 2012, Dong et al. showed that calcitriol protected renovascular function in hypertensive patients by downregulating angiotensin II type 1 receptor in endothelium cells (34). In addition, Li et al. demonstrated that VDR knockout mice showed an increased renin expression and hypertension, and these changes were suppressed by an analog of vitamin D (35). Vitamin D replacement restored MAP, RVR, RBF, and aldosterone plasma levels observed in our VDD+IRI+R rats in comparison to the IRI rats. Hence, our data reinforce the important role of vitamin D in blood pressure control.

A major feature of AKI is the presence of proteinuria. Hypovitaminosis D can exert important alterations in renal protein handling (36, 37). Studies have showed that VDD is associated with an increased prevalence of proteinuria, a marker of CKD progression, in adult population (36, 37). Our results showed a significant lower proteinuria in the VDD+IRI+R rats than in the VDD+IRI rats, showing that vitamin D replacement restored the renal protein handling in this group of animals. It is known that vitamin D levels can trigger proteinuria by direct or indirect factors. Directly, low levels of vitamin D can induce loss of podocytes and development of glomerulosclerosis, damaging the integrity of the glomerular filtration membrane (38). On the other hand, vitamin D suppresses renin transcription contributing to a reduction in proteinuria by hemodynamic effects (39). In 2008, Dusso et al. described that VDR is involved in the regulation of the RAAS, playing the role of a downregulator of this system (40). In our study, we observed a higher renal VDR expression in the VDD+IRI+R group provided by the restoration of vitamin D levels, which can explain at least in part, the lower proteinuria observed in these animals.

Expectedly, we observed higher plasma levels of PTH in the VDD+IRI rats. On the other hand, we found lower levels of this hormone in both IRI and VDD+IRI+R rats. In addition, we found an increased FEP in both VDD+IRI and VDD+IRI+R groups. It has been well-described that decreased plasma levels of vitamin D cause an elevation in plasma levels of PTH, leading to an increased urinary excretion of phosphorus (41). PTH inhibits phosphorus reabsorption in the kidney proximal tubule by the degradation of the co-transporter Na-Pi in the luminal membrane (41). In our study, it was noteworthy that the restoration of vitamin D levels, although not significant, was able to decrease FEP in the VDD+IRI+R rats in comparison to the VDD+IRI rats. Interestingly, our results demonstrated higher plasma levels of FGF-23 in the VDD+IRI+R rats. This result suggests that the restoration of vitamin D levels normalized plasma PTH levels in an attempt to decrease FEP, probably at the expense of higher plasma levels of FGF-23. FGF-23 is a phosphatonine that acts in the kidney to increase phosphorus excretion and decrease 1,25(OH)_2_D_3_ production (42). Moreover, some findings have supported an increasingly and important role of FGF-23 as an initial event in the development of CKD (3, 42). However, with the progression of kidney disease, a reduction in the levels of 1,25(OH)_2_D_3_ persists in addition to a secondary increase in PTH levels, leading to the development of secondary hyperparathyroidism (43). Thus, based on our results, we could infer that the restored levels of vitamin D acted on the PTH-vitamin D-FGF-23 axis, retarding renal disease progression.

Klotho is a potent therapeutic target regarding the different stages of CKD and a decline of its expression has been reported during renal disease progression (44, 45). It is known that renal I/R injury is related to Klotho deficiency (46). Previously, we demonstrated a reduction in Klotho expression in rats under VDD euthanized 60 days after I/R injury (4). Here, we also found a lower renal Klotho expression in the VDD+IRI rats. Conversely, our data showed that the restoration of vitamin D levels increased Klotho protein expression in VDD+IRI+R rats, followed by a similar profile of *klotho* gene expression. Klotho was identified as an aging suppressor gene in mouse (24). Moreover, Klotho is a single-pass membrane protein, mainly expressed in the kidney, acting as a co-receptor for FGF-23 (7, 24). This protein regulates direct and indirectly PTH production through the modulation of the plasma levels of 1,25(OH)_2_D_3_, phosphorus and FGF-23. Also, such alterations in vitamin D, PTH and FGF-23 levels are usually followed by a progressive decreased Klotho protein in urine of CKD patients (7, 24). Our data showed that the restoration of vitamin D levels ameliorated the renal Klotho expression in the VDD+IRI+R rats.

The pathological syndrome of CKD is characterized by a chronic reduction in kidney function and structural kidney damage (31, 47). The initial renal injury results in renal morphological changes such as necrosis, cast formation, tubular collapse and dilatation, inflammatory cell infiltrate and fibrosis (3). In previous studies, we found such alterations associated with an enlargement of the tubulointerstitial compartment in Nx and I/R rats. Furthermore, we showed that vitamin D deficiency *per se* or associated with Nx or I/R exacerbated the renal morphological alterations (3, 4). It is known that vitamin D is a renoprotector factor, however, the protective mechanisms of this hormone are still unclear (48). In the present study, we found higher expansion of the renal interstitial area associated with histological alterations (fibrosis, tubular atrophy and dilatation, and inflammatory cell infiltrate) in VDD+IRI rats. These morphological alterations were attenuated by the restoration of vitamin D levels in the VDD+IRI+R rats. Further, we analyzed the expression of two fibrous ECM components (fibronectin and COL3), the amount of MCP1, as well as the expression of CD68+ (macrophages) and CD3+ (T cells) cells in the renal tissue. Vitamin D deficiency increased the expression of both ECM markers, MCP1 amounts, and CD68+ and CD3+ cells expression in the renal tissue of VDD+IRI rats. In contrast, the restoration of vitamin D levels reestablished the amounts of COL3 and MCP1 as well as the renal expression of fibronectin, CD68+ and CD3+ cells in the VDD+IRI+R rats in comparison to the IRI rats. Macrophages are heterogeneous and their phenotype and functions are regulated by the surrounding micro-environment (49). Broadly, macrophages commonly exist in two different subtypes: classically activated or M1 macrophages, which are considered pro-inflammatory due to their capacity to release cytokines such as IL-1β, IL-6, IL-12, IL-23, and TNF-α; and alternatively activated or M2 macrophages, which are anti-inflammatory, immunomodulatory, and polarized by Th2 cytokines (3, 49, 50). It is known that M1/M2 macrophages balance regulates the inflammation as well as the tissue repair of an organ under injury (3, 49, 50). In 2018, we showed that the expression of macrophages (M1+M2) was increased in vitamin D-deficient rats submitted to Nx. In that same study, we found that VDD also reduced the expression of CD206+ cells (3). Here, we found similar results for CD68+ (M1+M2) cells in VDD+IRI rats. As mentioned earlier, the restoration of vitamin D levels decreased the expression of macrophages (CD68+ cells) as a whole in VDD+IRI+R rats. Although not statistically significant, it is important to highlight that the population of M2 macrophages was proportionally restored by vitamin D replacement in VDD+IRI+R rats. Despite M2 macrophages are considered to be pro-fibrotic, they also exert an important role in tissue repair, especially in acute and active renal lesions (3). Corroborating our data, Zhang et al. demonstrated that vitamin D prevented podocyte injury *via* regulation of macrophages M1/M2 phenotype in diabetic nephropathy rats (51). Thus, our results show that the restoration of vitamin D levels confirms the renoprotective effects of this hormone on the modulation of the expression of both ECM components and inflammatory cells.

Fibrosis and inflammation are hallmark features of CKD. Physiologically, renal fibrosis is a common downstream event in response to the initial renal insult (6, 52). However, unresolved renal inflammation turns into a major driving force to promote renal scar formation *via* a progressive process of RFF (6, 52). TGF-β is a multifunctional cytokine generally considered a potent pro-fibrotic mediator regarding CKD progression (26). Some studies have shown that vitamin D has proven to have beneficial effects in animal models as well as in clinical trials with chronic renal insufficiency, resulting in attenuation of renal fibrosis and kidney dysfunction (6). In order to clarify the effects of vitamin D in RFF, we analyzed the link between renal TGF-β1 and VDR expression. In 2014, we observed that VDD caused a decrease in VDR expression and an increase in TGF-β1 expression in vitamin D-deficient rats submitted to renal I/R injury (4). In this present study, we found similar results for both markers in the VDD+IRI rats. On the other hand, we observed a higher VDR expression associated with a restored expression of TGF-β1 caused by vitamin D replacement in the VDD+IRI+R rats in comparison to the IRI rats. These results were accompanied by a smaller FIA in the VDD+IRI+R group. Corroborating our data, Tan et al. showed that paricalcitol, a vitamin D analog, attenuated renal interstitial fibrosis in the obstructive nephropathy model. According to the authors, such improvement was due to the restoration of VDR expression and subsequent suppression of TGF-β expression and blockage of the epithelial–mesenchymal transition (EMT) (17). Thus, our data allowed us to infer that sufficient levels of vitamin D could help to slow the RFF probably due to an increase in VDR expression and a restoration of TGF-β expression in the kidneys of VDD+IRI+R rats.

The presence of epithelial cell phenotype modification to assume more mesenchymal characteristics is an integral part of tissue fibrogenesis after renal damage (53, 54). Previously, our research group demonstrated that vitamin D-deficient rats submitted to I/R or Nx models presented higher expression of vimentin and α-SMA in the renal cortex. According to those findings, we demonstrated that vitamin D was involved in the process of cellular phenotypic alteration (3, 4). In the present study, we observed that the restoration of vitamin D levels reestablished the renal expression of vimentin and α-SMA in the VDD+IRI+R rats in comparison to the IRI rats. A joint analysis of our data allows us to note that the restoration of vitamin D levels not only increased VDR expression but also reestablished TGF-β1, vimentin, and α-SMA expression in the renal tissue from VDD+IRI+R rats. Based on that, we could infer that those events observed in the renal cortex of VDD+IRI+R rats were possibly related to the smaller FIA as a result of an attenuation in the RFF. In 2012, Xiong et al. demonstrated that a loss of VDR in unilateral ureteral obstruction was considered a potential mechanism linking inflammation to EMT. According to the authors, this suppression on the VDR expression could lead the tubular epithelial cells to EMT and RFF induced by TGF-β and downregulation of β-catenin signaling (55). Therefore, our data highlight the importance of sufficient levels of vitamin D in maintaining VDR expression and its influence on the modulation of RFF and EMT.

Finally, we verified the contribution of the restoration of vitamin D levels on the improvement of the integrity of glomerular vascular endothelium in our renal disease model. In 2018, our group reported a lower expression of JG12 in glomerular capillaries in 5/6-nephrectomized rats under vitamin D deficiency (3). The JG12 protein is an aminopeptidase responsible for anchoring the cells in the cell membrane and it is considered as a specific marker for vascular endothelium (3, 56). The damage caused to the vascular endothelium is critical and triggers a series of pathological changes present in the progression of kidney disease (3, 56). Recently, we demonstrated that vitamin D deficiency contributed to vascular damage in ischemic-AKI model (12). Kumar et al. showed that the correction of vitamin D deficiency by cholecalciferol supplementation exerted a beneficial effect on vascular function in patients with early CKD (57). Here, we found that vitamin D deficiency decreased JG12 expression in the glomerular capillaries in VDD+IRI rats. Conversely, JG12 expression in the glomerular capillaries was improved by the restoration of vitamin D levels in VDD+IRI+R rats. Therefore, vitamin D replacement after I/R insult in rats previously deficient in vitamin D enhanced the expression of JG12 in the glomerular vascular endothelium, which could have probably contributed to the improvement of the renal function observed in these animals.

In conclusion, our data reinforce that vitamin D deficiency is an aggravating factor for renal diseases. Most important, the simple restoration of vitamin D levels by diet ameliorated the renal function, reestablished the hemodynamic parameters, and attenuated the tubulointerstitial damage (inflammation and fibrosis) in I/R rats previously deficient in vitamin D. The observation of vitamin D status as well as its replacement should be considered in the mitigation of renal disease progression.

## Data Availability Statement

The original contributions generated for the study are included in the article/supplementary material, further inquiries can be directed to the corresponding author/s.

## Ethics Statement

The animal study was reviewed and approved by Research Ethics Committee of Faculty of Medicine, University of São Paulo (CEUA, registration 1023/2018).

## Author Contributions

MdS, DC, DB, MS, AS, RV, and AdB: conceived and designed the experiments. MdS, DC, DB, MS, RV, and AdB: performed the experiments. MdS, DC, RV, and AdB: analyzed the data and contributed to the writing of the manuscript MdS, DC, RV, and AdB. All authors reviewed the manuscript.

## Conflict of Interest

The authors declare that the research was conducted in the absence of any commercial or financial relationships that could be construed as a potential conflict of interest.

## References

[1] Luyckx VA Cherney DZI Bello AK. Preventing CKD in Developed Countries. Kidney Int Rep. (2020) 5:263–77. 10.1016/j.ekir.2019.12.003 32154448 PMC7056854

[2] Luyckx VA Tuttle KR Garcia-Garcia G Gharbi MB Heerspink HJL Johnson DW. Reducing major risk factors for chronic kidney disease. Kidney Int Suppl. (2011) 7:71–87 (2017). 10.1016/j.kisu.2017.07.003 30675422 PMC6341126

[3] de Braganca AC Canale D Goncalves JG Shimizu MHM Seguro AC Volpini RA. Vitamin D deficiency aggravates the renal features of moderate chronic kidney disease in 5/6 nephrectomized rats. Front Med (Lausanne). (2018) 5:282. 10.3389/fmed.2018.00282 30370270 PMC6194324

[4] Goncalves JG de Braganca AC Canale D Shimizu MH Sanches TR Moyses RM. Vitamin D deficiency aggravates chronic kidney disease progression after ischemic acute kidney injury. PLoS ONE. (2014) 9:e107228. 10.1371/journal.pone.0107228 25222475 PMC4164619

[5] Kim HS Chung W Kim S. Vitamin D, and kidney disease. Electrolyte Blood Press. (2011) 9:1–6. 10.5049/EBP.2011.9.1.1 21998600 PMC3186891

[6] Lucisano S Buemi M Passantino A Aloisi C Cernaro V Santoro D. New insights on the role of vitamin D in the progression of renal damage. Kidney Blood Press Res. (2013) 37:667–78. 10.1159/000355747 24356557

[7] Patel TV Singh AK. Role of vitamin D in chronic kidney disease. Semin Nephrol. (2009) 29:113–21. 10.1016/j.semnephrol.2009.01.004 19371802 PMC2696155

[8] Dusso AS Brown AJ Slatopolsky E. Vitamin D. Am J Physiol Renal Physiol. (2005) 289:F8–28. 10.1152/ajprenal.00336.2004 15951480

[9] Dusso AS Tokumoto M. Defective renal maintenance of the vitamin D endocrine system impairs vitamin D renoprotection: a downward spiral in kidney disease. Kidney Int. (2011) 79:715–29. 10.1038/ki.2010.543 21270766

[10] Sassi F Tamone C D'Amelio P. Vitamin D: nutrient, hormone, and immunomodulator. Nutrients. (2018) 10:1656. 10.3390/nu10111656 30400332 PMC6266123

[11] de Braganca AC Volpini RA Canale D Goncalves JG Shimizu MH Sanches T. Vitamin D deficiency aggravates ischemic acute kidney injury in rats. Physiol Rep. (2015) 3:e12331. 10.14814/phy2.12331 25780095 PMC4393165

[12] de Braganca AC Volpini RA Mehrotra P Andrade L Basile DP. Vitamin D deficiency contributes to vascular damage in sustained ischemic acute kidney injury. Physiol Rep. (2016) 4:e12829. 10.14814/phy2.12829 27369932 PMC4945834

[13] Canale D de Braganca AC Goncalves JG Shimizu MH Sanches TR Andrade L. Vitamin D deficiency aggravates nephrotoxicity, hypertension and dyslipidemia caused by tenofovir: role of oxidative stress and renin-angiotensin system. PLoS ONE. (2014) 9:e103055. 10.1371/journal.pone.0103055 25048368 PMC4105615

[14] Petchey WG Johnson DW Isbel NM. Shining D' light on chronic kidney disease: mechanisms that may underpin the cardiovascular benefit of vitamin D. Nephrology (Carlton). (2011) 16:351–67. 10.1111/j.1440-1797.2011.01450.x 21323790

[15] Tamez H Kalim S Thadhani RI. Does vitamin D modulate blood pressure? Curr Opin Nephrol Hypertens. (2013) 22:204–9. 10.1097/MNH.0b013e32835d919b 23299053 PMC3984388

[16] Li YC. Vitamin D regulation of the renin-angiotensin system. J Cell Biochem. (2003) 88:327–31. 10.1002/jcb.10343 12520534

[17] Tan X Li Y Liu Y. Paricalcitol attenuates renal interstitial fibrosis in obstructive nephropathy. J Am Soc Nephrol. (2006) 17:3382–93. 10.1681/ASN.2006050520 17082242

[18] Kienreich K Tomaschitz A Verheyen N Pieber T Gaksch M Grubler MR. Vitamin D and cardiovascular disease. Nutrients. (2013) 5:3005–21. 10.3390/nu5083005 23912328 PMC3775239

[19] Megyesi J Andrade L Vieira JM Safirstein RLJ Price PM. Positive effect of the induction of p21WAF1/CIP1 on the course of ischemic acute renal failure. Kidney Int. (2001) 60:2164–72. 10.1046/j.1523-1755.2001.00044.x 11737590

[20] Sutton TA Molitoris BA. Mechanisms of cellular injury in ischemic acute renal failure. Semin Nephrol. (1998) 18:490–7. 9754601

[21] Burnette WN. “Western blotting”: electrophoretic transfer of proteins from sodium dodecyl sulfate—polyacrylamide gels to unmodified nitrocellulose and radiographic detection with antibody and radioiodinated protein A. Anal Biochem. (1981) 112:195–203. 10.1016/0003-2697(81)90281-5 6266278

[22] Lancas T Kasahara DI Gross JL Pires-Neto RC Deheinzelin D Mauad T. Cholinergic hyperresponsiveness of peripheral lung parenchyma in chronic obstructive pulmonary disease. Respiration. (2011) 82:177–84. 10.1159/000326897 21576920

[23] Livak KJ Schmittgen TD. Analysis of relative gene expression data using real-time quantitative PCR and the 2(-Delta Delta C(T)) method. Methods. (2001) 25:402–8. 10.1006/meth.2001.1262 11846609

[24] Kuro-o M. Klotho. Pflugers Arch. (2010) 459:333–43. 10.1007/s00424-009-0722-7 19730882

[25] Huen SC Cantley LG. Macrophages in renal injury and repair. Annu Rev Physiol. (2017) 79:449–69. 10.1146/annurev-physiol-022516-034219 28192060

[26] Sureshbabu A Muhsin SA Choi ME. TGF-beta signaling in the kidney: profibrotic and protective effects. Am J Physiol Renal Physiol. (2016) 310:F596–606. 10.1152/ajprenal.00365.2015 26739888 PMC4824143

[27] Schnaper HW. The Tubulointerstitial Pathophysiology of Progressive Kidney Disease. Adv Chronic Kidney Dis. (2017) 24:107–116. 10.1053/j.ackd.2016.11.011 28284376 PMC5351778

[28] Powers JG Gilchrest BA. What you and your patients need to know about vitamin D. Semin Cutan Med Surg. (2012) 31:2–10. 10.1016/j.sder.2011.11.008 22361283

[29] de Araujo M Andrade L Coimbra TM Rodrigues ACJ Seguro AC. Magnesium supplementation combined with N-acetylcysteine protects against postischemic acute renal failure. J Am Soc Nephrol. (2005) 16:3339–49. 10.1681/ASN.2004100832 16177005

[30] de Boer IH Katz R Chonchol M Ix JH Sarnak MJ Shlipak MG. Serum 25-hydroxyvitamin D and change in estimated glomerular filtration rate. Clin J Am Soc Nephrol. (2011) 6:2141–9. 10.2215/CJN.02640311 21836148 PMC3359004

[31] Webster AC Nagler EV Morton RL Masson P. Chronic kidney disease. Lancet. (2017) 389:1238–1252. 10.1016/S0140-6736(16)32064-5 27887750

[32] Zand L Kumar R. The use of vitamin D metabolites and analogues in the treatment of chronic kidney disease. Endocrinol Metab Clin North Am. (2017) 46:983–1007. 10.1016/j.ecl.2017.07.008 29080646 PMC5977979

[33] Vaidya A Williams JS. The relationship between vitamin D and the renin-angiotensin system in the pathophysiology of hypertension, kidney disease, and diabetes. Metabolism. (2012) 61:450–8. 10.1016/j.metabol.2011.09.007 22075270 PMC3290690

[34] Dong J Wong SL Lau CW Lee HK Ng CF Zhang L. Calcitriol protects renovascular function in hypertension by down-regulating angiotensin II type 1 receptors and reducing oxidative stress. Eur Heart J. (2012) 33:2980–90. 10.1093/eurheartj/ehr459 22267242

[35] Li YC Kong J Wei M Chen ZF Liu SQ Cao LP. 1,25-Dihydroxyvitamin D(3) is a negative endocrine regulator of the renin-angiotensin system. J Clin Invest. (2002) 110:229–38. 10.1172/JCI15219 12122115 PMC151055

[36] de Boer IH Ioannou GN Kestenbaum B Brunzell JD Weiss NS. 25-Hydroxyvitamin D levels and albuminuria in the Third National Health and Nutrition Examination Survey (NHANES III). Am J Kidney Dis. (2007) 50:69–77. 10.1053/j.ajkd.2007.04.015 17591526

[37] Lee DR Kong JM Cho KI Chan L. Impact of vitamin D on proteinuria, insulin resistance, and cardiovascular parameters in kidney transplant recipients. Transplant Proc. (2011) 43:3723–9. 10.1016/j.transproceed.2011.08.081 22172835

[38] Kuhlmann A Haas CS Gross ML Reulbach U Holzinger M Schwarz U. 1,25-Dihydroxyvitamin D3 decreases podocyte loss and podocyte hypertrophy in the subtotally nephrectomized rat. Am J Physiol Renal Physiol. (2004) 286:F526–33 10.1152/ajprenal.00316.2003 14600034

[39] Freundlich M Quiroz Y Zhang Z Zhang Y Bravo Y Weisinger JR. Suppression of renin-angiotensin gene expression in the kidney by paricalcitol. Kidney Int. (2008) 74:1394–402. 10.1038/ki.2008.408 18813285

[40] Dusso AS Brown AJ. Mechanism of vitamin D action and its regulation. Am J Kidney Dis. (1998) 32(2 Suppl. 2), S13-24. 10.1053/ajkd.1998.v32.pm9808140 9808140

[41] Moe SM. Disorders involving calcium, phosphorus, and magnesium. Prim Care. (2008) 35:215–37, v–vi. 10.1016/j.pop.2008.01.007 18486714 PMC2486454

[42] Christov M Waikar SS Pereira RC Havasi A Leaf DE Goltzman D. Plasma FGF23 levels increase rapidly after acute kidney injury. Kidney Int. (2013) 84:776–85. 10.1038/ki.2013.150 23657144 PMC3766419

[43] Quarles LD. Role of FGF23 in vitamin D and phosphate metabolism: implications in chronic kidney disease. Exp Cell Res. (2012) 318:1040–8. 10.1016/j.yexcr.2012.02.027 22421513 PMC3336874

[44] John GB Cheng CY Kuro-o M. Role of Klotho in aging, phosphate metabolism, and CKD. Am J Kidney Dis. (2011) 58:127–34. 10.1053/j.ajkd.2010.12.027 21496980 PMC3191324

[45] Kuro-o M. Klotho, phosphate and FGF-23 in ageing and disturbed mineral metabolism. Nat Rev Nephrol. (2013) 9:650–60. 10.1038/nrneph.2013.111 23774819

[46] Hu MC Shi M Zhang J Quinones H Griffith C Kuro-o M. Klotho deficiency causes vascular calcification in chronic kidney disease. J Am Soc Nephrol. (2011) 22:124–36. 10.1681/ASN.2009121311 21115613 PMC3014041

[47] Campanholle G Ligresti G Gharib SA Duffield S. Cellular mechanisms of tissue fibrosis. 3. Novel mechanisms of kidney fibrosis. Am J Physiol Cell Physiol. (2013) 304:C591–603. 10.1152/ajpcell.00414.2012 23325411 PMC3625718

[48] Arfian N Muflikhah K Soeyono SK Sari DC Tranggono U Anggorowati N. Vitamin D attenuates kidney fibrosis *via* reducing fibroblast expansion, inflammation, and epithelial cell apoptosis. Kobe J Med Sci. (2016) 62:E38-44. 27578035 PMC5425134

[49] Shapouri-Moghaddam A Mohammadian S Vazini H Taghadosi M Esmaeili SA Mardani F. Macrophage plasticity, polarization, and function in health and disease. J Cell Physiol. (2018) 233:6425–40. 10.1002/jcp.26429 29319160 PMC13482560

[50] Guiteras R Flaquer M Cruzado JM. Macrophage in chronic kidney disease. Clin Kidney J. (2016) 9:765–71. 10.1093/ckj/sfw096 27994852 PMC5162417

[51] Zhang XL Guo YF Song ZX Zhou M. Vitamin D prevents podocyte injury *via* regulation of macrophage M1/M2 phenotype in diabetic nephropathy rats. Endocrinology. (2014) 155:4939–50. 10.1210/en.2014-1020 25188527

[52] Gu YY Liu XS Huang XR Yu XQ Lan HY. Diverse role of TGF-beta in kidney disease. Front Cell Dev Biol. (2020) 8:123. 10.3389/fcell.2020.00123 32258028 PMC7093020

[53] Ferenbach DA Bonventre JV. Mechanisms of maladaptive repair after AKI leading to accelerated kidney ageing and CKD. Nat Rev Nephrol. (2015) 11:264–76. 10.1038/nrneph.2015.3 25643664 PMC4412815

[54] Nogueira A Pires MJ Oliveira PA. Pathophysiological mechanisms of renal fibrosis: a review of animal models and therapeutic strategies. In Vivo. (2017) 31:1–22. 10.21873/invivo.11019 28064215 PMC5354133

[55] Xiong M Gong J Liu Y Xiang R Tan X. Loss of vitamin D receptor in chronic kidney disease: a potential mechanism linking inflammation to epithelial-to-mesenchymal transition. Am J Physiol Renal Physiol. (2012) 303:F1107–15. 10.1152/ajprenal.00151.2012 22791341

[56] Li P Ma LL Xie RJ Xie YS Wei RB M. Yin. Treatment of 5/6 nephrectomy rats with sulodexide: a novel therapy for chronic renal failure. Acta Pharmacol Sin. (2012) 33:644–51. 10.1038/aps.2012.2 22555371 PMC4010349

[57] Kumar V Yadav AK Lal A Kumar V Singhal M Billot L. A Randomized trial of vitamin D supplementation on vascular function in CKD. J Am Soc Nephrol. (2017) 28:3100–3108. 10.1681/ASN.2017010003 28667080 PMC5619968

